# Butyrate inhibits *Staphylococcus aureus*-aggravated dermal IL-33 expression and skin inflammation through histone deacetylase inhibition

**DOI:** 10.3389/fimmu.2023.1114699

**Published:** 2023-05-16

**Authors:** Chia-Hui Luo, Alan Chuan-Ying Lai, Ya-Jen Chang

**Affiliations:** ^1^ Institute of Biomedical Sciences, Academia Sinica, Taipei, Taiwan; ^2^ Taiwan International Graduate Program in Molecular Medicine, National Yang Ming Chiao Tung University and Academia Sinica, Taipei, Taiwan; ^3^ Department of Pharmaceutical Sciences, School of Pharmacy, College of Pharmacy, Taipei Medical University, Taipei, Taiwan; ^4^ Institute of Translational Medicine and New Drug Development, China Medical University, Taichung, Taiwan

**Keywords:** atopic dermatitis, butyrate, histone deacetylase, interleukin 33, keratinocytes, S. aureus, S. epidermidis

## Abstract

Atopic dermatitis (AD) is an inflammatory skin disease caused by the disruption of skin barrier, and is dominated by the type 2 immune responses. Patients with AD have a high risk of developing *Staphylococcus aureus* infection. Interleukin-33 (IL-33), an alarmin, has been implicated in the pathophysiology of AD development. Butyrate, a short chain fatty acid known to be produced from the fermentation of glycerol by the commensal skin bacterium, *Staphylococcus epidermidis*, has been reported to possess antimicrobial and anti-inflammatory properties that suppress inflammatory dermatoses. However, little is known about the effects of butyrate on dermal IL-33 expression and associated immune response in *S. aureus*-aggravated skin inflammation in the context of AD. To decipher the underlying mechanism, we established an AD-like mouse model with epidermal barrier disruption by delipidizing the dorsal skin to induce AD-like pathophysiology, followed by the epicutaneous application of *S. aureus* and butyrate. We discovered that *S. aureus* infection exacerbated IL-33 release from keratinocytes and aggravated dermal leukocyte infiltration and IL-13 expression. Moreover, we showed that butyrate could attenuate *S. aureus*-aggravated skin inflammation with decreased IL-33, IL-13, and leukocyte infiltration in the skin. Mechanistically, we demonstrated that butyrate suppressed IL-33 expression and ameliorated skin inflammation through histone deacetylase 3 (HDAC3) inhibition. Overall, our findings revealed the potential positive effect of butyrate in controlling inflammatory skin conditions in AD aggravated by *S. aureus* infection.

## Introduction

AD is a chronic, relapsing, and inflammatory skin condition with a complex etiology. It is characterized by epidermal thickening and dermal leukocyte infiltration and is mediated by epithelial-derived IL-33 and Th2 cytokines, including IL-4, IL-5, and IL-13 (1, 2). Studies have reported that secretion of IL-33 by epithelial cells during cellular injury or damage activates type 2 innate lymphoid cells (ILC2), T helper 2 cells (Th2), macrophages, and eosinophils to generate a Th2-skewed microenvironment (3), leading to the development of Th2 inflammation. To study the disease mechanisms of AD, the establishment of an AD-like mouse model is essential. Recent studies have suggested that skin delipidized by organic solvent mixture (AEW, acetone/ether, and water washing protocol) results in hyper-proliferation of keratinocytes in the basal layer and scratching, the cardinal feature of AD (4, 5).

The human skin microbiota harbors a plethora of resident microorganisms of different species. These microorganisms can interact with host cells, as well as the host immune system (3). One such microbe is *S. aureus*, which is a gram-positive bacterium residing in skin and mucosa. The superficial skin is a major site for *S. aureus* colonization, which normally resides in 10-20% of healthy individuals (6). Moreover, studies have reported that 80-100% of AD patients are colonized with *S. aureus*, which is strongly associated with disease severity (7, 8). *S. aureus* is capable of secreting virulence factors, including biofilm, enterotoxins, and superantigens that are considered as important elements of the vicious cycle of AD (9). Importantly, a disrupted skin barrier increases the susceptibility to *S. aureus* infection in skin lesions, which is associated with the overexpression of Th2 cytokines in AD patients (8, 10).

Short-chain fatty acids (SCFAs) are microbiota-derived metabolites composed of a carboxylic acid moiety and a hydrocarbon tail. SCFAs are produced by microbes in the gut and by skin-commensal bacteria on the skin at low concentrations (11, 12). Studies have shown that SCFAs derived from skin-commensal bacterium *S. epidermidis* inhibit the overgrowth of an opportunistic pathogen on the skin, *Propionibacterium acnes* (13, 14). Moreover, the products of *S. epidermidis* can suppress cutaneous inflammation through a mechanism depending on the toll-like receptor TLR2 (15). SCFAs reportedly also influenced inflammatory disease symptoms. For example, SCFAs could mediate cytokine and chemokine production by leukocytes and epithelial cells through the activation of G-protein-coupled receptors GPR41 and GPR43 and the inhibition of histone deacetylases (HDACs) (16). However, the regulatory role of SCFAs on IL-33 expression and skin inflammation in the context of *S. aureus* infection remains poorly understood.

As a previous study has demonstrated that SCFA butyrate, produced from the glycerol fermentation of *S. epidermidis* in the skin microbiome, could inhibit *S. aureus* growth (17), and our own previous study on butyrate alone has demonstrated its ability to regulate type 2 innate lymphoid cell function in the context of airway inflammation and hyperreactivity (18), we choose to focus on butyrate to investigate its regulatory role in *S. aureus*-aggravated skin inflammation.

## Materials and methods

### Mice

Three-week-old C57BL/6 mice were purchased from National Laboratory Animal Center (Taipei, Taiwan) and housed under specific pathogen-free conditions.

### Bacterial strains

*S. epidermidis* (ATCC 12228) and AD patients-isolated *S. aureus* were kindly provided by Prof. Chun-Ming Huang (University of California, San Diego, USA) (17). The bacteria were cultured in tryptic soy broth (TSB, Sigma-Aldrich) at 37 ˚C with 250 rpm shaking overnight. Aliquots of *S. aureus* culture were centrifuged at 2200 ×*g*, and the pellets were washed with phosphate-buffered saline (PBS) and adjusted to an optical density of 1 at 600 nm, corresponding to approximately 1 × 10^9^ *S. aureus* cells/mL.

### Cell lines

KERTr cells were purchased from the American Type Culture Collection (Manassas, VA, USA) and cultured in antibiotic-free keratinocyte-SFM medium (Thermo Fisher).

### Establishment of an AD-like mouse model, infection with *S. aureus*, and butyrate treatment

Hair from the dorsal skin (1.0 × 1.0 cm^2^) of C57BL/6 mice was shaved prior to the start of the experiment. To perform the delipidization treatment, the organic solvent of acetone and ether mixture (1:1) was applied to the shaved area twice daily for two days (4, 5). For epicutaneous *S. aureus* infection, 5 × 10^8^ colony-forming units (CFU) of *S. aureus* was suspended in 0.1 mL of TSB and applied to a cotton pad (1.0 × 1.0 cm^2^), which was then applied onto the dorsal skin of mice for 24 h following delipidization. For intradermal *S. aureus* infection, C57BL/6 mice were intradermally (*i.d.*) inoculated into their backs with 10^7^ colony-forming units (CFU) of *S. aureus* suspended in 0.1 mL of PBS and were sacrificed three days after *S. aureus* inoculation. For butyrate treatment on *S. aureus*-infected skin, after delipidization treatment, the dorsal skin of C57BL/6 mice was treated with a cotton pad (1.0 × 1.0 cm^2^) for 24 h with 5 × 10^8^ CFU of *S. aureus* that was suspended in 0.1 mL of 1 mM butyrate.

### Treatment of *S. epidermidis* glycerol ferment on mouse skin

Glycerol fermentation by *S. epidermidis* was performed using a method described in a previous study (17). In brief, C57BL/6 mice subjected to AEW treatment were treated with 50 ul of *S. epidermidis* or *S. aureus* (5 × 10^8^ CFU) individually or simultaneously, in the presence or absence of 2% glycerol, using a cotton pad (1.0 × 1.0 cm^2^). After 24 h of infection, the dorsal skin of the mice was excised and cut into smaller pieces for cytokine detection.

### Mouse skin thickness measurement and histology

Mice dorsal skin was excised and fixed with 4% formaldehyde (Merck) for 24 h, and gradually dehydrated with 50%, 70%, and 90% ethanol (J.T. Baker) for 15 minutes each. The skin samples were embedded in paraffin for further histological analysis. For epidermal thickness, the skin sections were stained with hematoxylin and eosin (H&E) and measured using an Olympus CX31 microscope (Olympus Corp, Tokyo, Japan).

### Processing of mouse skin and preparation of single-cell suspension

Dorsal skin (1.0 × 1.0 cm^2^) was excised from delipidized mice, cut into smaller pieces, and incubated in 5 mL DMEM containing 1 mg/mL DNase I (Worthington Biochemicals), 0.5 mg/mL collagenase type I (Worthington Biochemicals), and 0.2 mg/mL collagenase type II (Worthington Biochemicals) for 60 min at 37°C. The digested tissue suspension was then filtered through a 70-μm mesh to obtain a single-cell suspension. The red blood cells in the single-cell suspension were lysed using ACK lysing buffer (Thermo Fisher) before the cells were resuspended in the appropriate buffer for further processing.

### Isolation and culturing of murine primary keratinocytes

The murine primary keratinocytes were isolated following an established protocol (19). Briefly, neonates from C57B/6 mice aged 0-2 days were sacrificed, and the limbs removed just above the wrist and ankle joints. The whole skin was then peeled off and incubated in 4.3 mg/mL neutral protease at 4 ˚C overnight. The next day, forceps were used to separate the epidermis from the dermis, which was then further digested using 0.25 mg/mL trypsin/EDTA to obtain a single suspension of keratinocytes. Freshly isolated keratinocytes were cultured in antibiotic-free progenitor cell targeted medium (CELLnTEC) in 96-well plates at a density of 1 × 10^5^ cells in 0.2 mL/well for further experimentation.

### *In vitro* infection with S. aureus

Cells were cultured at 1 × 10^5^ cells/mL in 96-well plates and infected with *S. aureus* at 10 MOI (multiplicity of infection) for 24 h. The culture supernatant was then collected for ELISA measurement. For mRNA detection, cells were cultured in antibiotic-free medium at 5 × 10^5^ cells/mL in 12-well plates and infected with *S. aureus* at 10 MOI prior to mRNA isolation.

### Determination of SCFA bactericidal activity

*S. aureus* (2×10^7^ CFU/mL) was incubated with PBS (control) or 1 mM of acetate, butyrate or propionate for 24 h. After incubation, a 10-fold serial dilution from 10^-1^ to 10^-5^ was prepared from the treated bacteria, and 5 μl of each dilution was spotted on an agar plate. The bactericidal activity of SCFA was then determined in CFU from the highest dilution that showed bacterial growth.

### Cytotoxicity analysis

KERTr cells (10^5^ cells/well) were treated with butyrate for 24 h, and the cytotoxicity of butyrate was evaluated using the MTT assay (**Supplementary Table 2**) according to the manufacturer’s instructions.

### Flow cytometry

Single cell suspensions were washed once with PBS and stained with 100 μL FVD (eBioscience) diluted in PBS for 30 min at 4°C. Cells were washed with equal volume of PBS/2% FCS, followed by Fc blocking with 100 μL anti-human TruStain FcX (BioLegend) in PBS/2% FCS for 10 min at 4°C. Cells were then stained with the PerCP/Cy5.5 anti-CD45 (Biolegend) for 30 min at 4°C and were washed with equal volume of PBS/2% FCS and resuspended in 200 μL PBS/2% FCS for FACS analysis. Data were acquired using an LSR II (BD Biosciences) and analyzed using FlowJo v.10.1 software (TreeStar).

### ELISA

The dorsal skin (1.0 × 1.0 cm^2^) was cut into smaller pieces and transferred to 1 mL of DMEM. The explants were then incubated at room temperature with gentle shaking for 30 min. After incubation, the supernatant from the organ culture was collected and assayed for cytokines using ELISA kits according to the manufacturer’s instructions. The ELISA kits used for cytokine detection are listed in **Supplementary Table 1**.

### Immunoblotting

KERTr cells (2×10^6^ cells/well) were lysed in protein lysis buffer, sonicated, and centrifuged at 13,000 ×*g* for 10 min at 4 ˚C to remove cell debris. Protein concentration was determined using the micro-BCA protein assay kit (ThermoFisher). Equal amounts of protein (12 µg/sample) were separated using SDS-PAGE and transferred to PVDF membranes (PALL Corporation). Membranes were blocked with Tris-buffered saline and Tween 20 containing 5% bovine serum albumin. The primary antibodies for immunoblotting are listed in **Supplementary Table 2**. The primary antibodies were detected with peroxidase-conjugated anti-rabbit IgG or anti-mouse IgG, followed by detection with Western Lightning ECL pro reagent (PerkinElmer) according to the manufacturer’s recommendations.

### Immunofluorescence staining of skin tissue sections

Mouse dorsal skin was harvested, fixed with 4% formaldehyde (Merck), and gradually dehydrated with 50%, 70%, and 90% ethanol (J.T. Baker) for 15 min each. Paraffin-embedded skin sections were stained with streptavidin Alexa Fluor 488-conjugated anti mouse K14 antibody, Alexa Fluor 568-conjugated anti mouse HDAC2 antibody, and Alexa Fluor 568-conjugated anti mouse HDAC3 antibody (**Supplementary Table 1**), followed by the Alexa Fluor-conjugated secondary antibody (Thermo Fisher). Tissue auto-fluorescence was reduced by incubating slides with 1% Sudan black (Sigma-Aldrich) for 20 min before mounting. Image acquisition was performed using an Olympus BX51 microscope.

### Quantitative real-time-PCR

Total RNA was isolated from skin tissue or cultured cells and extracted using the Quick-RNA™ MicroPrep (Zymo Research) according to the manufacturer’s instructions. A total of 1-2 μg of cDNA was synthesized using the high-capacity cDNA reverse transcription kit (Applied Biosystems), and real-time PCR was conducted using an Optical 96 real-time PCR thermal cycler (Biometra). Reactions were run in triplicate, and samples were normalized to *GAPDH* expression and quantities determined according to the 2^-ΔΔCt^ method. The primers used for qRT-PCR are listed in **Supplementary Table 3**.

### Statistical analysis

Statistical analyses were performed using Prism 6 (GraphPad Prism). Student’s unpaired *t*-test or one‐way ANOVA were used to determine statistical significance between groups; values of *p* < 0.05 were considered significant.

## Results

### *S. aureus* infection induces the expression of epithelial cell-derived IL-33 *in vitro*

**Figure 1:**
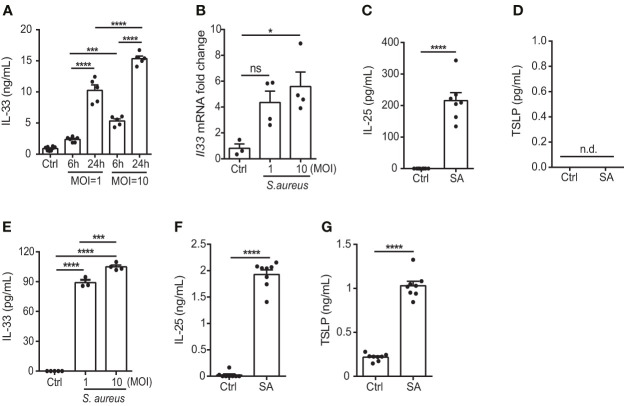
*S. aureus* infection induces the expression of epithelial cell-derived IL-33 *in vitro*. **(A)** IL-33 protein levels in KERTr cells infected with *S. aureus* at multiplicity of infection (MOI) of 1 and 10 for 6 h and 24 h. **(B)** IL-33 mRNA levels in KERTr cells infected with *S. aureus* at MOI of 1 and 10 for 6 h. **(C, D)**. Protein levels of **(C)** IL-25 and **(D)** TSLP secreted by KERTr cell under *S. aureus* infection at MOI of 10. **(E–G)**. Protein levels of **(E)** IL-33, **(F)** IL-25, and **(G)** TSLP secreted by mouse primary keratinocytes under *S. aureus* infection at MOI of 10. Data are shown as mean ± SEM from 3 independent experiments (n=3-8 per group). Statistical analysis was performed using one-way ANOVA **(A, B, E)** or an unpaired two-tailed t test **(C, D, F, G)**. *n.d,* Not detectable; *n.s,* Not significant. *p<0.05, ***p<0.001, ****p<0.0001.

*S. aureus* is a common opportunistic pathogen associated with chronic inflammation in patients with skin disorders such as AD (20); however, its effect on the function of keratinocytes remains unclear. Therefore, to elucidate the potential role of *S. aureus* in contributing to the exacerbation of allergic responses during AD, we tested the ability of a *S. aureus* strain isolated from an AD patient to induce IL-33, as well as two other epithelial cell-derived cytokines, IL-25 and thymic stromal lymphopoietin (TSLP), in the human keratinocyte cell line, KERTr cell. We found that the *S. aureus* clinical isolate triggered the release of IL-33 in an infection dose- and time-dependent manner (**Figures 1A, B**). Moreover, IL-25 also increased in *S. aureus*-infected KERTr cells (**Figure 1C**). However, TSLP was undetectable in *S. aureus* infected KERTr cells (**Figure 1D**). A similar effect was observed in primary mouse keratinocytes infected with *S. aureus*, in which increased IL-33 and IL-25 expression was observed, along with positive TSLP expression (**Figures 1E–G**).

### Epicutaneous exposure of *S. aureus* aggravates IL-33 release and skin inflammation

**Figure 2:**
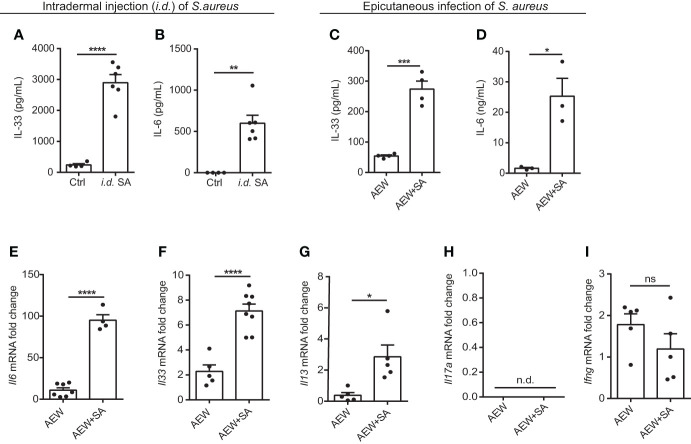
Epicutaneous exposure of *S. aureus* aggravates IL-33 release and skin inflammation. (**A, B**) Three-week old C57BL/6 mice were intradermally (*i.d.*) inoculated with 10^7^ colony-forming units (CFU) of *S. aureus* and sacrificed 3 days after *S. aureus* infection. **(A)** IL-33 protein level in the skin. **(B)** IL-6 protein level in the skin. **(C–I).** Three-week old C57BL/6 mice were treated with the AEW protocol daily for 2 days, followed by the epicutaneous *S. aureus* infection at 5 × 10^8^ CFU for 24 h. **(C)** IL-33 protein level in the skin. **(D)** IL-6 protein level in the skin. **(E–I).** mRNA levels of **(E)** *Il6*, **(F)** *Il33*, **(G)** *Il13*, **(H)** *Il17a*, and **(I)** *Ifng* in the skin. Data are shown as mean ± SEM from 3 independent experiments (n=3-8 per group). Statistical analysis was performed using an unpaired two-tailed t test **(A–I)**. *n.d.* Not detectable. *n.s.* Not significant. *p<0.05, **p<0.01, ***p<0.001, ****p<0.0001.

To determine whether *S. aureus* infection could induce IL-33 expression *in vivo*, we infected C57BL/6 mice with *S. aureus* by intradermal injection (*i.d.*). We observed an increase in IL-33 and IL-6 production from *S. aureus*-infected mice (**Figures 2A, B**). As a recent study has reported that *S. aureus* could penetrate through the epidermis into the dermis and trigger the expression of Th2 cytokines (21), we established an AD-like mouse model to mimic the natural route of *S. aureus* infection in AD patients with skin barrier disruption. This involved applying AEW treatment to mice, followed by epicutaneously challenging them with *S. aureus.* We discovered that *S. aureus* infection exacerbated AEW-induced IL-33 and IL-6 expressions in the skin (**Figures 2C, D**). Furthermore, the mRNA levels of *Il6* and *Il33*, as well as the Th2 cytokine *Il13*, increased substantially in *S. aureus*-infected skin (**Figures 2E–G**). However, the level of Th17 cytokine *Il17a* mRNA was undetectable (**Figure 2H**), and the level of Th1 cytokine *Ifng* mRNA did not change significantly during *S. aureus* infection (**Figure 2I**). Overall, these results suggest that *S. aureus* infection enhances IL-33 induction and aggravates Th2 inflammation in the skin of mice.

### *S. epidermidis* glycerol ferment suppresses *S. aureus*-aggravated IL-33

**Figure 3:**
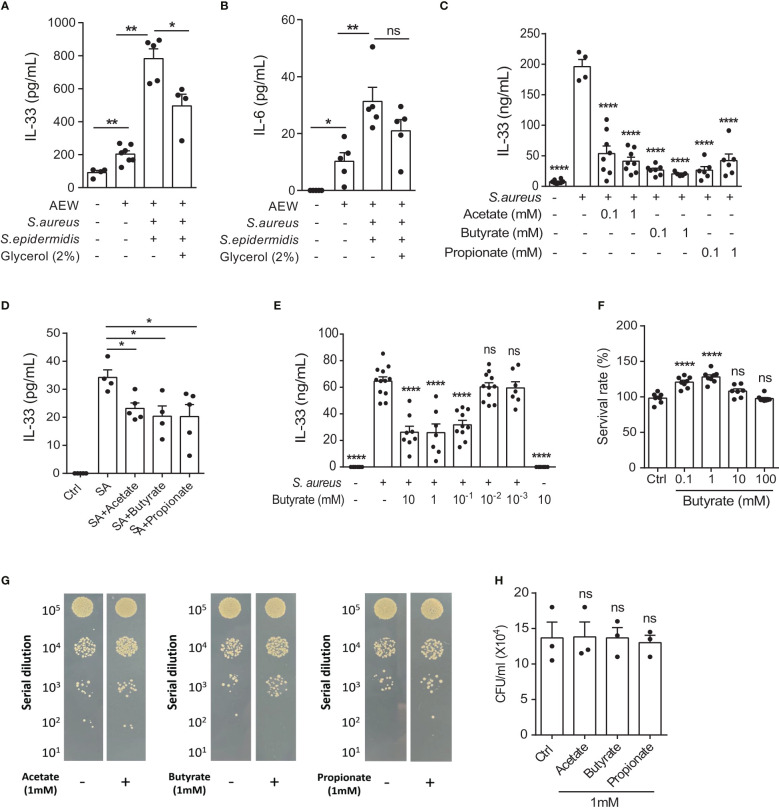
*S. epidermidis* glycerol ferment suppresses *S. aureus*-aggravated IL-33. **(A, B)**. Three-week old C57BL/6 mice were first subjected to AEW treatment twice daily for 2 days, and were then epicutaneously challenged with *S. epidermidis* or *S. aureus* (5 × 10^8^ CFU) individually or simultaneously, with or without 2% glycerol. Mice were sacrificed the next day following the epicutaneous challenges **(A)** IL-33 level in the skin. **(B)** IL-6 level in the skin. **(C, D)**. IL-33 secreted by **(C)** KERTr cells and **(D)** mouse primary keratinocytes infected with *S. aureus* and treated concurrently with acetate, butyrate, or propionate for 24 h. **(E)** Level of IL-33 from KERTr cell infected with *S. aureus* and treated with butyrate for 24 h. **(F)** Survival rate of KERTr cells after 24 h of butyrate treatment. **(G, H)**. The evaluation *S. aureus* (2×10^7^ CFU/ml) growth after incubation with 1 mM of acetate, butyrate, or propionate for 24 h. Data are shown as mean ± SEM from 3 independent experiments (n=3-8 per group). Statistical analysis was performed using one-way ANOVA (A-F and H). *n.s,* Not significant. *p<0.05, **p<0.01, ****p<0.0001.

Previous studies have reported that SCFAs possess antimicrobial and anti-inflammatory properties, suggesting immunomodulatory roles (17, 22). Therefore, we examined whether *S. epidermidis* grown in 2% glycerol could suppress *S. aureus*-induced IL-33 and IL-6 *in vivo*. C57BL/6 mice subjected first to AEW treatment were epicutaneously challenged with *S. epidermidis* or *S. aureus* (5 × 10^8^ CFU), individually or simultaneously, with or without 2% glycerol. We found that the IL-33 level was reduced when the skin was treated with the two bacteria in the presence of glycerol compared with the treatment with the two bacteria without glycerol (**Figure 3A**). However, the expression of IL-6 was not significantly altered by the addition of 2% glycerol (**Figure 3B**).

Three SCFAs, acetate, butyrate, and propionate, are known to be produced by the fermentation of glycerol by *S. epidermidis* (23). Consequently, we investigated the effect of all three SCFAs on cultured keratinocytes following *S. aureus* infection. We found that all three SCFAs could inhibit IL-33 expression in the KERTr cells (**Figure 3C**), as well as in the mouse primary keratinocytes (**Figure 3D**). Since butyrate treatment in KERTr cells at 1mM demonstrated a trend of stronger IL-33 suppression compared to the other two SCFAs, we further examined the dose-response of butyrate treatment on IL-33 expression. We found that butyrate suppressed IL-33 expression in *S. aureus*-infected KERTr cells in a dose-dependent manner (**Figure 3E**), with the IL-33 suppression effect plateauing at 1 mM butyrate. We also assessed the toxicity effect of butyrate treatment on KERTr cells and observed no reduction in viability in butyrate-treated KERTr cell at the concentrations we examined, even at a high concentration of 100 mM (**Figure 3F**). Although all three SCFAs could inhibit IL-33 expression in *S. aureus*-infected keratinocytes, they did not suppress the growth of *S. aureus* (**Figures 3G, H**). This suggests that the inhibition of IL-33 in *S. aureus*-infected keratinocytes was not due to a reduction of *S. aureus* viability, but rather resulted from the regulatory effect of SCFAs on IL-33 expression.

### Butyrate suppresses IL-33 production and attenuates skin inflammation

**Figure 4:**
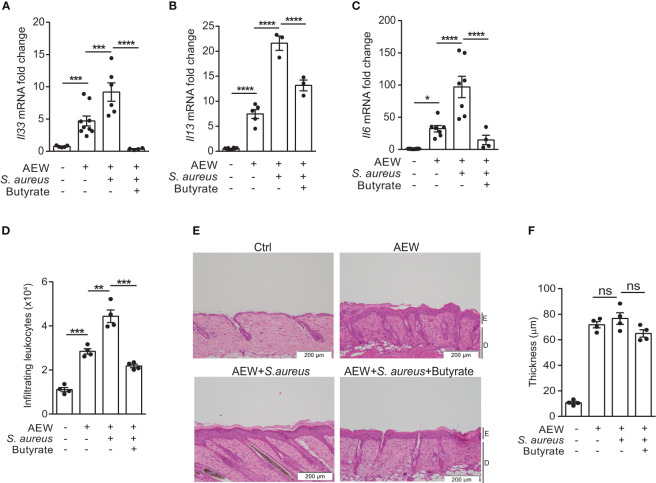
Butyrate suppresses IL-33 production and attenuates skin inflammation. **(A–F)**. *S. aureus* (5×10^8^ CFU) was applied onto the dorsal skin of mice with or without butyrate (1 mM) after the last AEW treatment. Mice were sacrificed the next day for further experimentation. **(A–C)**. Levels of **(A)** *Il33*, **(B)** *Il13*, and **(C)** *Il6* mRNAs in the skin. **(D)** Mean numbers of total CD45^+^ leukocytes in the skin. **(E)** Hematoxylin and eosin (H&E) stained skin sections. (Scale bars: 200 μm, Magnification: 10X; E, epidermidis; D, dermal). **(F)** Measurement of skin thickness. Data are shown as mean ± SEM from 3 independent experiments (n=3-9 per group). Statistical analysis was performed using one-way ANOVA **(A–C, E–F)**. *n.s,* Not significant. *p<0.05, **p<0.01, ***p<0.001, ****p<0.0001.

Encouraged by the results of our previous study on using butyrate as an immunomodulatory agent (18), we turned our focus to assess the effects of butyrate on *S. aureus*-induced skin inflammation. We concurrently applied *S. aureus* and butyrate to the dorsal skin after AEW treatment, and the expression levels of *Il33*, *Il13*, and *Il6* mRNA were diminished by butyrate co-treatment (**Figures 4A–C**). Simultaneously, we found that butyrate decreased the number of dermal infiltrating leukocytes in *S. aureus*-infected mice (**Figure 4D**; **Supplemental Figure 1**). However, the epidermal thickening was not influenced by butyrate treatment (**Figure 4E**). Collectively, these results demonstrated that butyrate could inhibit *S. aureus*-exacerbated skin inflammation by suppressing IL-33 release from keratinocytes.

### Butyrate suppresses IL-33 expression in *S. aureus*-infected KERTr cell through HDAC3 inhibition

**Figure 5:**
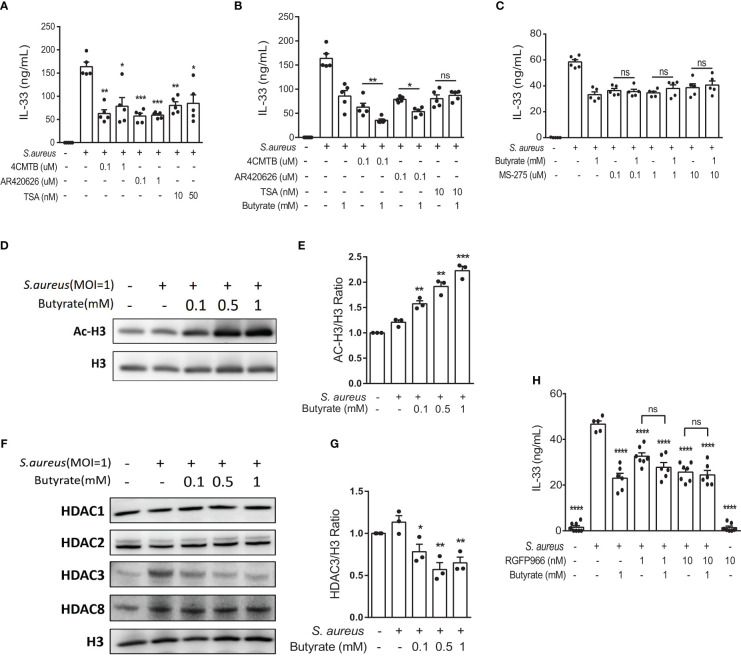
Butyrate suppresses IL-33 expression in *S. aureus*-infected KERTr cells through HDAC3 inhibition. **(A)** IL-33 level in the culture supernatant of KERTr cells infected with *S. aureus* in conjunction with the treatment of 4-CMTB (0.1 and 1 µM), AR420626 (0.1 and 1 µM), or TSA (10 and 50 nM). **(B)** IL-33 level in the culture supernatant of KERTr cells infected with *S. aureus* in conjunction with the treatment of 4-CMTB (0.1 µM), AR420626 (0.1 µM), or TSA (10 nM), and in the presence or absence of butyrate (1 mM). **(C)** IL-33 level in the culture supernatant of KERTr cells infected with *S. aureus* in conjunction with the treatment of MS-275 (0.1, 1, and 10 µM), and in the presence or absence of butyrate (1 mM). **(D–G)**. KERTr cells infected with *S. aureus* were treated with butyrate (0.1, 0.5, and 1 mM) for 6 h. **(D)** Representative western blot of acetylated histone 3 (Ac-H3) and total histone 3 (H3). **(E)** Quantification of Ac-H3 normalized to H3. **(F)** Representative western blot of class I HDACs (HDAC1, 2, 3, and 8) and H3. **(G)** Quantification of HDAC3 normalized to H3. **(H)** IL-33 level in the culture supernatant of KERTr cells infected with *S. aureus* and treated RGFP966 (1 and 10 nM) in the presence or absence of butyrate (1 mM) 24 h. Data are shown as mean ± SEM from 3 independent experiments (n=3-7 per group). Statistical analysis was performed using one-way ANOVA **(A–C, E, G, H)**. *n.s,* Not significant. *p<0.05, **p<0.01, ***p<0.001, ****p<0.0001, compared to *S. aureus* infection alone groups.

Previous studies have shown that SCFAs exert modulatory functions either by binding to free fatty acid receptors FFAR2 and FFAR3 to activate downstream signaling pathways (24, 25) or by inducing histone acetylation through HDAC inhibitory activity (26–28). To determine the involvement of FFAR2, FFAR3, and HDACs in response to butyrate in *S. aureus* infection, we first treated *S. aureus*-infected KERTr cells in the absence of butyrate with FFAR2 agonist 4-chloro-α-(1-methylethyl)-N-2-thiazolyl-benzeneacetamide (4-CMTB), the FFAR3 agonist AR420626, or the pan-HDAC inhibitor trichostatin A (TSA). We found that both receptor agonists and the pan-HDAC inhibitor suppressed *S. aureus-*induced IL-33 production (**Figure 5A**). Next, to clarify the mechanism underlying IL-33 release, KERTr cells were infected with *S. aureus* and treated with butyrate alone or co-treated with butyrate in the presence of 4-CMTB, AR420626, or TSA. As expected, the results showed that both FFAR2 and FFAR3 agonists synergized with butyrate to inhibit IL-33 expression (**Figure 5B**). However, the effect of butyrate and TSA co-treatment on the level of IL-33 level did not differ significantly from either treatment on their own (**Figure 5B**).

The expression of HDAC3, a member of the class I HDAC family, is known to correlate with the expression of IL-33 (29). Therefore, we assessed the possible involvement of HDAC3 in IL-33 expression in *S. aureu*s-infected keratinocytes. We found that the class I HDAC inhibitor MS-275 alone suppressed *S. aureus-*induced IL-33 production in KERTr cells, and the co-treatment of butyrate and MS-275 did not further downregulate IL-33 expression in comparison to either treatment alone (**Figure 5C**), suggesting that butyrate might act as a HDAC inhibitor and lead to increased histone acetylation and regulated gene transcription. We went on to measure the level of histone 3 acetylation (AC-H3) using western blot and found that butyrate treatment caused an increase in histone 3 acetylation in KERTr cells in a dose-dependent manner (**Figures 5D, E**).

We next investigated whether butyrate acted on HDAC targets similarly to MS-275 in *S. aureu*s-infected KERTr cells. Western blot analysis for class I HDACs HDAC1, 2, 3, and 8 revealed that only HDAC3 levels were reduced by butyrate treatment (**Figures 5F, G**; **Supplemental Figures 2A–C**). To further determine the involvement of HDAC3 in IL-33 expression, we treated *S. aureus*-infected KERTr cells with HDAC3 antagonist RGFP966. We found that co-treatment with butyrate and RGFP966 did not further suppress IL-33 expression compared to either treatment alone (**Figure 5H**). Collectively, our data suggest that butyrate can inhibit IL-33 expression in *S. aureus*-infected KERTr cells by blocking HDAC3 action.

### Butyrate ameliorates skin inflammation induced by *S. aureus* through HDAC3 inhibition

**Figure 6:**
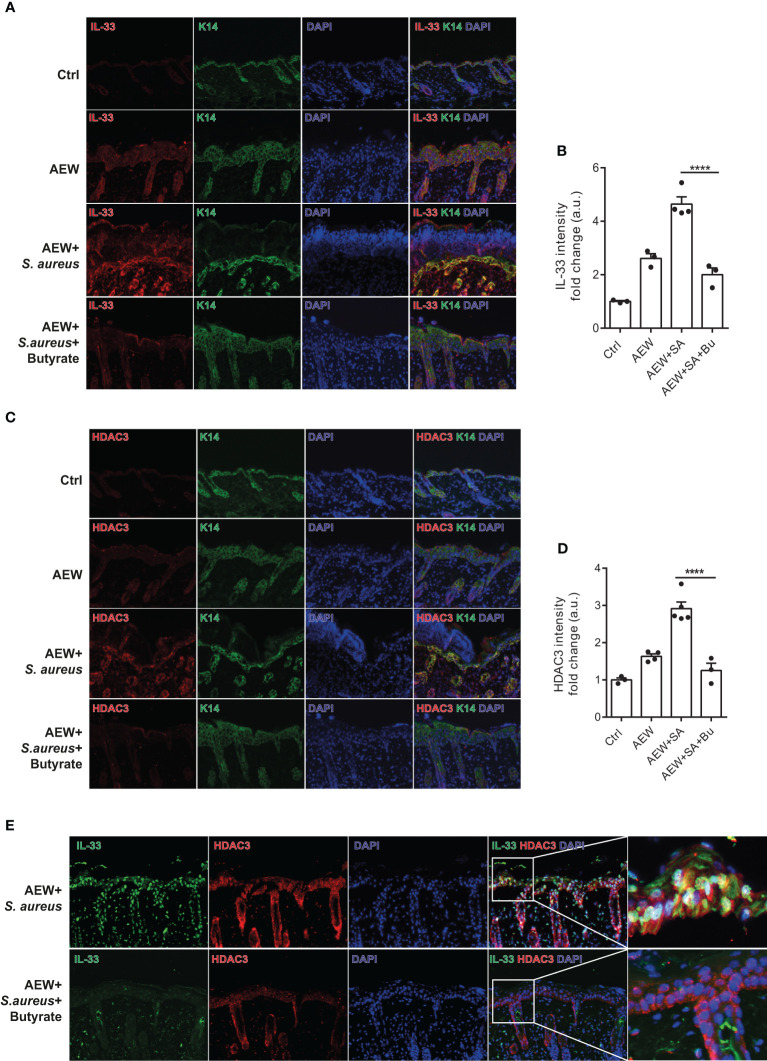
Butyrate ameliorates skin inflammation induced by *S. aureus* through HDAC3 inhibition. **(A)** Immunofluorescence staining of IL-33 (red), K14 (green), and DAPI (blue) in the skin of WT mice. (Magnification: 40X). **(B)** Densitometry analysis for IL-33 in the mouse skin. **(C)** Immunofluorescence staining of HDAC3 (red), K14 (green), and DAPI (blue) in the skin of WT mice. (Magnification: 40X). **(D)** Densitometry analysis for HDAC3 in the mouse skin. **(E)** Immunofluorescence staining of IL-33 (green), HDAC3 (red), and DAPI (blue) in the skin of WT mice. (Magnification: 40X). Data are shown as mean ± SEM from 3 independent experiments (n=3-5 per group). Statistical analysis was performed using one-way ANOVA **(B, D)**. ****p<0.0001.

We performed additional analysis on the protein expression of IL-33 in the skin of WT mice using immunofluorescence staining. Our results revealed that *S. aureus* infection led to increased IL-33 expression in the delipidized skin. Nevertheless, treatment with butyrate significantly reduced *S. aureus*-aggravated IL-33 expression in the skin (**Figures 6A, B**). To investigate the effect of butyrate on HDAC3 expression, we found that butyrate treatment significantly reduced *S. aureus*-exacerbated HDAC3 expression in the skin (**Figures 6C, D**). However, we did not observe a significant difference in the expression of other members of the HDAC class I, such as HDAC2, between the groups (**Supplemental Figures 3A, B**). We also observed a reduction in the co-localization of HDAC3 and IL-33 in butyrate-treated skin (**Figure 6E**). This suggests that butyrate inhibits keratinocyte-derived IL-33 expression in *S. aureus*-infected skin cells through HDAC3 inhibition.

## Discussion

**Figure 7:**
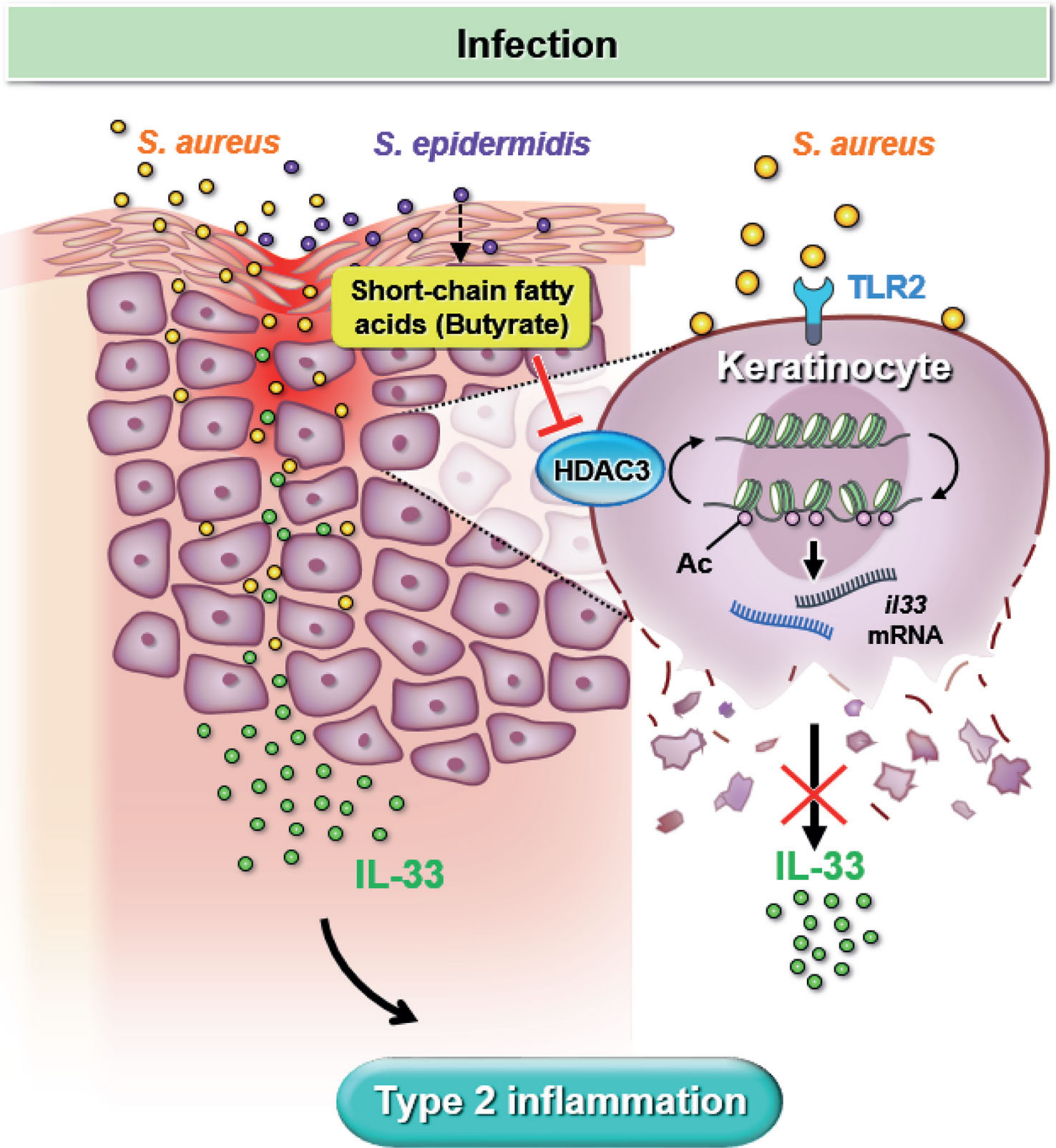
Butyrate attenuates *S. aureus*-aggravated dermal IL-33 and skin inflammation through histone deacetylase 3 inhibition. *S. aureus* infection exacerbates keratinocyte-derived IL-33 expression and contributes to skin inflammation. Butyrate suppresses *S. aureus*-aggravated dermal IL-33 expression to ameliorate skin inflammation through HDAC3 inhibition.

Our study utilized both *in vitro* experiments and *in vivo* mouse models to simulate *S. aureus* infection in dry skin condition experienced by AD patients. We demonstrated that *S. aureus* infection could worsen skin inflammation by increasing IL-33 release from keratinocyte and exacerbate dermal leukocyte infiltration. Furthermore, we elucidated the regulatory role of butyrate, a metabolite of skin commensal bacterium *S. epidermidis*, which can attenuate skin inflammation resulting from *S. aureus* infection. Mechanistically, we found that butyrate could suppress keratinocyte-derived IL-33 expression in *S. aureus*-infected KERTr cells by inhibiting HDAC3. Overall, our findings, illustrated in **Figure 7**, suggest that butyrate may serve as a therapeutic agent for treating inflammatory skin conditions in AD patients with *S. aureus* infection.

Skin commensal bacteria are known to produce the SCFA butyrate, which has been shown to prevent skin inflammatory response by activating skin resident regulatory T cells (30). SCFAs also inhibit Th2 responses and interact with other immune cells (31). In our prior study, we have demonstrated that butyrate, but not acetate or propionate, inhibits the Th2 response mediated by murine ILC2s in the lungs (18). Thus, based on our previous experience with SCFAs, we selected butyrate to investigate its effect on *S. aureus*-aggravated inflammation in the skin. Our results show that butyrate attenuates skin inflammatory responses, as evidenced by decreased infiltrating leukocytes and IL-33 expression both *in vitro* and *in vivo*. Although a previous study has reported that a high concentration of 50 mM butyrate is required to suppresses the growth of *S. aureus* (17),, we indirectly confirmed this by demonstrating that no suppression of *S. aureus* growth was observed at the high dosage of 1 mM SCFAs (**Figures 3G, H**). Therefore, directly using butyrate to suppress *S. aureus* growth on the skin of AD patients may not be feasible. However, our data also showed that as little as 0.1 mM of butyrate was enough to inhibit *S. aureus*-aggravated IL-33 expression *in vitro* (**Figures 3C, E**), suggesting butyrate as a potential therapeutic agent for *S. aureus*-exacerbated skin inflammation.

In addition, our *in vitro* experiment also shows that acetate and propionate, likewise produced by the *S. epidermidis* glycerol fermentation (23), can inhibit IL-33 release from *S. aureus*-infected keratinocytes (**Figures 3C, D**). Therefore, we cannot exclude the possibility that acetate and propionate can also suppress IL-33 expression to ameliorate skin inflammation. Further investigation is needed to determine whether acetate or propionate has a similar inhibitory effect as butyrate on *S. aureus*-exacerbated skin inflammation *in vivo*. In short, our results suggest that microbial metabolites can positively influence *S. aureus*-exacerbated skin inflammation.

SCFAs produced by microbes have been reported to alter gene expression and cell proliferation by inhibiting the chromatin-remodeling activity of HDACs (26). In allergic skin inflammation, HDAC3 inhibition by TSA suppresses MCP1 expression, alleviating mast cell-mediated passive cutaneous anaphylaxis (32). Furthermore, butyrate-induced HDAC3 inhibition in the intestine leads to metabolic changes and the induction of the bactericidal function of macrophages, promoting gut homeostasis (33). In our investigation, we first established that butyrate could inhibit IL-33 expression in *S. aureus*-infected human keratinocyte KERTr cells through HDAC inhibition, especially of the class I HDAC. Subsequently, we demonstrated that butyrate upregulated histone 3 acetylation and downregulated HDAC3 expression. We also used a HDAC3-specific inhibitor in *S. aureus*-infected KERTr cells to clarify the role of HDAC3 in IL-33 regulation and determine the inhibitory function of butyrate on HDAC3 activity. In the *S. aureus*-infected skin, we found that butyrate treatment significantly reduced the co-expression of IL-33 and HDAC3-expressing cells, further suggesting the regulation of butyrate in HADC3 and IL-33 expression.

Our data suggest that butyrate acts as an inhibitor of the class I HDACs in *S. aureus*-infected KERTr cells, and the downregulation of HDAC3 by butyrate attenuated IL-33 expression. However, the downstream effects of HDAC inhibition remain to be elucidated. Although HDAC3 can act as a transcriptional repressor to influence IL-33 expression in many diseases, including multiple sclerosis and allergic asthma (29), it is still unclear whether the inhibitory effect of butyrate is due to the direct acetylation of the *Il33* gene or indirectly through the transcriptional activation/suppression of other targets that can alter the IL-33 expression. Further investigation is needed to understand the mechanism of butyrate’s inhibitory effect on IL-33 expression.

In summary, our model of epicutaneous application of *S. aureus* to delipidized skin mimicked the natural route of *S. aureus* infection in patients with AD. We demonstrated the regulatory role of butyrate, produced by the skin commensal bacterium *S. epidermidis*, in suppressing HDAC3 activity and attenuating skin inflammation caused by *S. aureus* infection. Additionally, we revealed the underlying mechanism of butyrate in regulating IL-33 and provided evidences supporting butyrate as a potential therapeutic agent for controlling inflammatory skin conditions exacerbated by *S. aureus* infection in AD patients.

## Data availability statement

The original contributions presented in the study are included in the article/**Supplementary Material**. Further inquiries can be directed to the corresponding authors.

## Ethics statement

The animal study was reviewed and approved by Academia Sinica Institutional Animal Care and Use Committee.

## Author contributions

Conceptualization, writing – original draft, and writing – review and editing, C-HL, AL, Y-JC. Methodology and visualization, C-HL. Investigation, C-HL and AL. Funding acquisition, project administration, and supervision, Y-JC. All authors contributed to the article and approved the submitted version.

## Supplementary material

Supplementary Figure 1 Butyrate reduces infiltrating dermal leukocytes in *S. aureus*-infected mice. Representative flow cytometry plot showing dermal leukocytes (FVD^+^ CD45^+^), assessed by FACS.

Supplementary Figure 2 Butyrate does not alter HDAC1, HDAC2, and HDAC8 expressions in KERTr cells under *S. aureus* infection. KERTr cells infected with *S. aureus* were treated with butyrate (0.1, 0.5, and 1 mM) for 6 h, and the ratios of **(A)** HDAC1, **(B)** HDAC2, and **(C)** HDAC8 normalized to total histone 3 were determined. Data are shown as mean ± SEM from 3 independent experiments (n=3-7 per group). Statistical analysis was performed using one-way ANOVA. *n.s.* Not significant, compared to *S. aureus* infection alone groups.

Supplementary Figure 3 Butyrate does not alter HDAC2 expression in the skin of mice with tested conditions under *S. aureus* infection. **(A)**. Immunofluorescence staining of HDAC2 (red), K14 (green), and DAPI (blue) in the skin of mice. (Magnification: 40X) **(B)**. Densitometry analysis for HDAC2 in the mouse skin. Data are shown as mean ± SEM from 3 independent experiments (n=3-7 per group). Statistical analysis was performed using one-way ANOVA **(B)**. *n.s.* Not significant, compared to *S. aureus* infection alone groups.
