# Supplementary material for: Butyrate inhibits Staphylococcus aureus-aggravated dermal IL-33 expression and skin inflammation through histone deacetylase inhibition
Source: Front Immunol. 2023 May 16;14:1114699. doi: 10.3389/fimmu.2023.1114699 (PMC10228744; doi:10.3389/fimmu.2023.1114699)
Supplement: Supplementary file 4 [file Table_1.docx]

**Supplementary Tables**

**Table S1. Antibodies used for immunoblotting and tissue staining.**

| **Antibody** | **Clone** | **Concentration**  **(μg/ml)** | **Supplier** |
| --- | --- | --- | --- |
| anti-Ac-H3 | N/A | WB (1:2000) | Cell Signaling |
| anti-HDAC1 | N/A | WB (1:1000) | Santa Cruz |
| anti-HDAC2 | N/A | WB (1:1000) | GeneTex |
| anti-HDAC3 | N/A | WB (1:1000) | GeneTex |
| anti-HDAC8 | N/A | WB (1:1000) | Santa Cruz |
| anti-H3 | N/A | WB (1:2000) | Cell Signaling |
| Anti-IL-33 | N/A | IF (1:200) | Thermofisher |
| Anti-HDAC2 | N/A | IF (1:200) | GeneTex |
| Anti-HDAC3 | N/A | IF (1:200) | GeneTex |
| Anti-K14 | N/A | IF (1:200) | Abcam |

**Table S2. Critical commercial assays**

| **Reagent** | **Supplier** | **Identifier** |
| --- | --- | --- |
| Mouse IL-33 ELISA Kit | R&D | Cat# DY3626-15 |
| Mouse IL-6 ELISA Kit | eBioscience | Cat# 88706488 |
| Mouse IL-25 ELISA Kit | eBioscience | Cat# 88700288 |
| Mouse TSLP ELISA Kit | eBioscience | Cat#88749088 |
| Human IL-33 ELISA Kit | PeproTech | Cat# 900-k398 |
| Human IL-6 ELISA Kit | BioLegend | Cat# 430501 |
| Human IL-25 ELISA Kit | R&D | Cat#DY125805 |
| Human TSLP ELISA Kit | eBioscience | Cat#88749788 |
| Butyrate | Sigma | Cat# 303410 |
| Acetate | Sigma | Cat# S5636 |
| Propionate | Sigma | Cat# P1880 |
| MTT Assay Kit | Abcam | Cat#ab211091 |

**Table S3. Primers used for qRT-PCR**

| **Gene** | **Species** | **Sequence (5’-3’)** |
| --- | --- | --- |
| *Gapdh* | mouse | Forward: AGGTCGGTGTGAACGGATTTG  Reverse: TGTAGACCATGTAGTTGAGGTCA |
| *Il6* | mouse | Forward: CAAAGCCAGATCAGA  Reverse: GATGGTCTTGGTCCTTAGCC |
| *Il33* | mouse | Forward: ATTTCCCCGGCAAAGTTCAG  Reverse: AACGGAGTCTCATGCAGTAGA |
| *Tslp* | mouse | Forward: AGGCTACCCTGAAACTGAG  Reverse: GGAGATTGCATGAAGGAATACC |
| *Il13* | mouse | Forward: CCTGGCTCTTGCTTGCCTT  Reverse: GGTCTTGTGTGATGTTGCTCA |
| *Ifng* | mouse | Forward: GGCCATCAGCAACAACATAAGCGT  Reverse: TGGGTTGTTGACCTCAAACTTGGC |
| *Il17a* | mouse | Forward: TCCAGAAGGCCCTCAGACTA  Reverse: ACACCCACCAGCATCTTCTC |
| *Gapdh* | human | Forward: AGGTCGGAGTCAACGGATTTG  Reverse: TGTAAACCATGTAGTTGAGGTC |
| *Il33* | human | Forward: CAAAGAAGTTTGCCCCATGT  Reverse: AAGGCAAAGCACTCCACAGT |
